# *Bacillus thuringiensis* Spores and Vegetative Bacteria: Infection Capacity and Role of the Virulence Regulon PlcR Following Intrahaemocoel Injection of *Galleria mellonella*

**DOI:** 10.3390/insects10050129

**Published:** 2019-05-05

**Authors:** Christophe Buisson, Michel Gohar, Eugénie Huillet, Christina Nielsen-LeRoux

**Affiliations:** Micalis Institute, INRA, AgroParisTech, Université Paris-Saclay, 78350 Jouy-en-Josas, France; christophe.buisson@inra.fr (C.B.); michel.gohar@inra.fr (M.G.); eugenie.huillet@inra.fr (E.H.)

**Keywords:** *Bacillus thuringiensis*, *Galleria mellonella*, haemocoel, spores, virulence, bio-pesticide, PlcR-regulon

## Abstract

*Bacillus thuringiensis* is an invertebrate pathogen that produces insecticidal crystal toxins acting on the intestinal barrier. In the *Galleria mellonella* larvae infection model, toxins from the PlcR virulence regulon contribute to pathogenicity by the oral route. While *B. thuringiensis* is principally an oral pathogen, bacteria may also reach the insect haemocoel following injury of the cuticle. Here, we address the question of spore virulence as compared to vegetative cells when the wild-type Bt407cry- strain and its isogenic ∆*plcR* mutant are inoculated directly into *G. mellonella* haemocoel. Mortality dose-response curves were constructed at 25 and 37 °C using spores or vegetative cell inocula, and the 50% lethal dose (LD_50_) in all infection conditions was determined after 48 h of infection. Our findings show that (i) the LD_50_ is lower for spores than for vegetative cells for both strains, while the temperature has no significant influence, and (ii) the ∆*plcR* mutant is four to six times less virulent than the wild-type strain in all infection conditions. Our results suggest that the environmental resistant spores are the most infecting form in haemocoel and that the PlcR virulence regulon plays an important role in toxicity when reaching the haemocoel from the cuticle and not only following ingestion.

## 1. Introduction

*Bacillus thuringiensis* strains are among the most widely used larvicidal entomopathogen [1]. *B. thuringiensis* is a member of the *Bacillus cereus* group, composed of several genetically closely related Gram positive and sporulating bacterial species [2,3], including the human opportunistic pathogen *B. cereus*, which is involved in food intoxication or more severe systemic infections [4]. *B. cereus* and *B. thuringiensis* are ubiquitously present in the environment. The major difference between *B. cereus* strains and *B. thuringiensis* is the presence of larvicidal crystal toxins in latter. The *cry* genes encoding these toxins are generally carried on large plasmids. Cry toxins are produced as inclusion bodies (“crystals”) during the bacterial sporulation phase. Insecticidal action is initiated after ingestion of inclusion bodies by a susceptible larva, in which they are solubilized by the alkaline pH of the midgut, resulting in release of the protoxin, which is next processed by digestive enzymes to its active form. The activated toxin binds to several receptors on the gut epithelial surface resulting in pore formation, gut paralysis, decreased food uptake, and final death of the larva (for review [5,6]). Although the high toxicity of Cry toxins make them the most important virulence factors produced by *B. thuringiensis*, other factors also take part in the infection process. Indeed, in some insects and nematodes, full virulence requires the presence of spores and germinated vegetative bacteria in addition to Cry toxins. This is the case for *Galleria mellonella*, the greater wax moth [7]. Therefore, this insect is currently used as a model to explore various aspects of the *B. thuringiensis* infection process, including virulence and adaptation effectors, regulation networks involved in the production of these effectors, and the bacterium life cycle within the insect [8,9,10,11,12]. In this model, *B. thuringiensis* or *B. cereus* may be directly injected into the haemocoel, or can be administered by oral infection (force-feeding). In force-feeding, a mixture of vegetative cells and Cry1Ca toxin act in synergy to induce high larval mortality, whereas neither the bacteria nor the Cry1Ca toxin alone affects the larvae [13]. The strength of this synergy model was further proven when the assay, run with the *∆plcR* mutants of Bt 407 (crystal minus) or *B. cereus* ATCC 14579 strains, resulted in a strong decrease of insect mortality as compared to the wild-type strains. The PlcR protein is a transcriptional regulator (originally named as regulator of the gene encoding the phopholipase C). PlcR is involved in activation and thus expression of a large number of secreted toxins and enzymes composing the PlcR regulon [14]. The PlcR regulon is required for bacterial pathogenicity, when spores are introduced via the oral route [13]. To be active, PlcR needs PapR, a signalling peptide, and these two factors comprise a quorum sensing system [15]. As observed with the *∆plcR* deletion strain, deletion of the *papR* gene strongly reduced virulence of *B. thuringiensis* strain 407cry- (crystal minus) in *G. mellonella* following oral infection [10]. Interestingly, virulence of the ∆*plcR* mutant was reportedly not reduced after injection into haemocoel, leading Salamitou et al. 2000 [13] to suggest that the PlcR regulon is not required for pathogenicity when spores are mechanically introduced into the haemocoel. However, other studies indicated that some PlcR factors are expressed in the *G. mellonella* haemocoel [9,12] and could be involved in infection, as reported by Bouillaut et al. 2005 [16]. In all previous virulence studies, using the *G. mellonella* model, infection tests by injection were either performed at 37 °C [11,17] or at 25°C [10,13,18], and most of the time the infectious dose was delivered as either spores or vegetative cells but not both. It is; therefore, of interest to compare these two forms in the same study.

In the context of transmission of pathogens from one host to another, understanding the transfer of infection is essential to elucidate pathogenesis and as part of the pathogen’s ecology. Horizontal transmission better fits to *B. thuringiensis* ecology than vertical transmission. Indeed, this pathogen kills its host and can grow and sporulate in the insect cadaver [9,11,19]. *B. thuringiensis* and to some extent *B. cereus* have a complex ecological cycle that may involve cycling between the insect larvae and the soil-plant environment [19,20,21,22], and the ecology of *B. thuringiensis* is still not fully understood [23]. 

Under natural conditions, the local density of spores and crystals can be low and for some strains the spore and crystal are separated following mother cell lysis. For these reasons, the probability to ingest spores and crystal together is likely to be low. In such conditions, and because the spore alone is inactive by the oral route, it might well be that infection from the cuticle, through spiracles or injury, has an underestimated role in *B. thuringiensis* persistence and transmission. Therefore, it is of relevance to further explore *B. thuringiensis* and *B. cereus* insect infection and virulence capacity, following haemocoel infection, which we here chose to do by performing experiments with the frequently used crystal minus *B. thuringiensis* strain, Bt407cry-.

This study has three objectives: (i) Evaluate and compare the virulence capacity of *B. thuringiensis* spores and vegetative cells following injection into the *G. mellonella* haemocoel; (ii) determine the impact of the infection temperature 37 versus 25 °C; and (iii) determine whether the PlcR regulon plays a role during haemocoel infection. The results should increase the knowledge of *B. thuringiensis* ecology and give clues on the relative role of secreted virulence factors from the PlcR regulon present in both *B. thuringiensis* and *B. cereus* strains. 

## 2. Materials and Methods

### 2.1. Bacterial Strains and Cultures

The study was carried out with the *B. thuringiensis* crystal minus strain Bt407cry- (referred to below as Bt407WT) and the interruption mutant Bt407cry- ∆*plcR*, (∆*plcR* mutant) as described in Salamitou et al. 2000 [13]. The ∆*plcR* mutant is resistant to kanamycin 200 µg/mL. *B. thuringiensis* cultures for vegetative cells were made in Luria-Bertani (LB) broth, incubated at 37 °C, in 1:10 volume liquid:air flasks at 175 rpm. The vegetative bacteria used for infection were recovered from mid log phase (Do_600_ = 1). Spore preparations for infection experiments were prepared in 100 mL (Hydrolysate of Casein and Tryptone) HCT -sporulation medium [24] in 1:10 volume liquid:air flasks at 175 rpm at 30 °C until complete spore formation and cell lysis (72 h). Spores were collected by centrifugation as described previously [13], and resuspended in sterile water at 1/10 of the initial volume, reaching about 3 × 10^7^ cfu (colony forming units)/mL, estimated following heating for 15 min at 78 °C and from serial dilutions on LB agar plates. 

### 2.2. Mortality Tests in Galleria mellonella

Haemocoel infection assays were performed with last instar *G. mellonella* larvae (250 mg), reared in our laboratory (INRA, Jouy-en-Josas or Versailles) on pollen and bee wax (La ruche Roannaise Besachier, Roanne, France). Larvae were injected using a syringe and needle with an injector pump KDS100 from KD Scientific, Thermo Fisher, Illkirch, France.

Each larva was inoculated with 10 µL suspension of vegetative bacteria or spores from Bt407WT or ∆*plcR* mutant strains. Mortality tests were performed using a range of doses (250–35,000 cfu per larva), appropriate to establish a full dose–response curve. Control larvae were injected with saline alone. A total of 25–30 larvae were infected per condition and tests were repeated at least three times. Larvae were incubated in small plastic dishes (5 per dish) without food at 25 or 37 °C. Mortality was scored at 48 hours post-treatment. Mortality rates were calculated by dividing the number of dead larvae by the number of total exposed larvae. 

### 2.3. Statistical Analysis

The dataset from larval mortality, obtained at 48 h, consisted of 131 observations obtained with the two strains (Bt407WT, ∆*plcR*), at 25 or 37 °C, and with spores or vegetative cells. Three to 4 biological replications and 4 to 5 doses were used per condition. The LD_50_ (dose which kills 50% of totally treated larvae) values were determined by non-linear regression using the statistical software JMP9 (SAS Institute Inc., Cary, NC, USA). The model used for the regression was a derivative of the Hill equation X^n^/X^n^+a^n^, where x is the dose used and “a” and “n” are the computed parameters. The parameters, determined with their 95% confidence intervals (CI_95_%), represent the LD_50_ (a) and the steepness of the curve (n). LD_50_s were considered as significantly different, with *p* < 0.05 for the null hypothesis, when the CI_95_% upper and lower confidence intervals did not overlap.

## 3. Results

### 3.1. Relative Roles of Temperature, Spores, and Vegetative Bacteria in Virulence Tests

**Figure 1:**
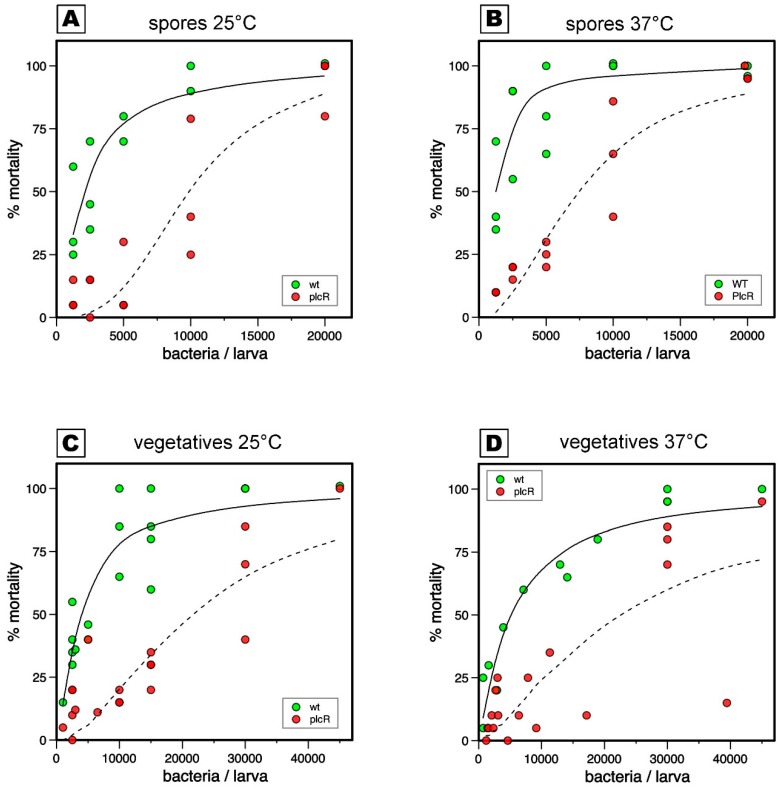
Dose–response mortality curves at 48 h. Larvae mortalities are plotted against bacterial doses and are shown as green dots (WT), Bt407WT, or as red dots, (*plcR*) ∆ *plcR* mutant. Non-linear regressions fitted to the experimental data are shown as plain lines (WT) or dotted lines *(plcR*). Mortalities at 48 h post-injection of spores at incubation temperature of 25 °C (**A**) or 37 °C (**B**). Mortalities post-injection of vegetative bacteria at 25 °C (**C**) or 37 °C (**D**).

**Figure 2:**
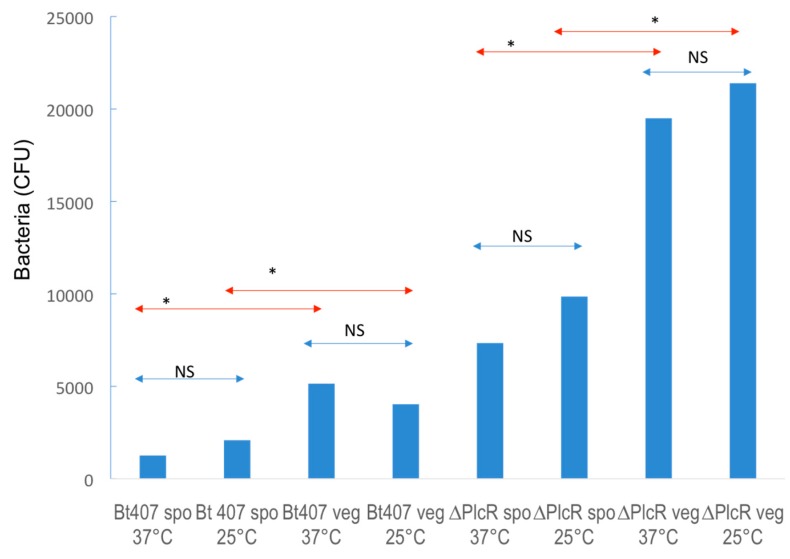
Temperature has no significant influence on the LD_50_. Comparisons between 25 and 37 °C of LD_50_ values (represented as double arrow lines). Y-axis Bacteria (dose in CFU: colony forming units), X-axis: various conditions). LD_50_ were considered as significantly different, with *p* < 0.05 for the null hypothesis, when the CI_95%_ did not overlap. Non-significant (NS) (blue); significant (* red).

Virulence results, 48 h after infection, from experiments performed on *G. mellonella* larvae with Bt407WT and ∆*plcR* mutant strains are presented in Figure 1 and Figure 2 and in Table 1 and Table 2. Figure 1 shows all the mortality data obtained with Bt407WT and *∆plcR* mutant strain spores at 25 and 37 °C (Figure 1A,B, respectively) and vegetative cells at 25 and 37 °C (Figure 1C,D, respectively). The LD_50_ of the two strains were estimated with their CI_95%_ for all mortality test conditions (Table 1 and Table 2). The LD_50_ of spores was about four times significantly (*p* < 0.05) lower than of the LD_50_ of vegetative cells at 37 °C, and about two times lower (*p* < 0.05) at 25 °C (Table 1 and Table 2, Figure 2). In contrast, no difference in LD_50_ was detected between 37 and 25 °C incubation conditions when tested with spores or vegetative cells (Table 1 and Table 2, Figure 2). Thus, the LD_50_ of both strains after *G.mellonella* intra-haemocoelic infection is independent of incubation conditions but dependent of the presence of spore or vegetative cell in the infectious dose.

### 3.2. The PlcR Regulon Is Required for Virulence When Spores or Vegetative Cells Are Mechanically Introduced into the Haemocoel

The LD_50_ of ∆*plcR* mutant strain was about five times higher than that of Bt407WT when spores were used for mortality tests at both 37 and 25 °C. (Table 1, Figure 1 and Figure 2). Additionally, the LD_50_ of the ∆*plcR* mutant was about four times higher than that of the wild-type strain when vegetative cells were used for mortality testing at both 37 and 25 °C. Therefore, in contrast with previous data [13], we found that the PlcR regulon is required for full Bt407WT virulence when spores or vegetative cells are mechanically introduced into the haemocoel. 

## 4. Discussion

One of the main issues of this study was to determine the relative infection capacity of spores versus vegetative bacteria from the Bt407WT strain after injection into the haemocoel of larvae of the insect *G. mellonella*. We also explored to what extent factors from the virulence regulon PlcR might be important for pathogenicity by this route*,* and if incubation temperature had an impact. To answer these questions, we set up a dose–response assay to calculate the different LD_5*0*_. Our results indicate that whatever the condition or the strain, spores display a significantly lower LD_50_ than vegetative cells. The LD_50_ ratios between spores and vegetative cells were, for the Bt407WT strain, four fold at 37 °C and two fold at 25 °C, while for the *∆plcR* mutant strain, the ratio was close to two at both temperatures. This indicates that under the conditions of our study, spores are more infective than vegetative bacteria for both strains. When comparing, Bt407WT and *∆plcR* stains, larval mortality was recorded at 24, 48, and 72 h post injection (data not shown for 24 and 72 h). We chose to show the data at 48 h only, as the plateau of the LD_50_ was reached at this time point. Indeed, at 24 h the ratios between the two strains was higher, but no difference was found between the strains at 48 and 72 h post injection. Therefore, is seems like at a given dose the *∆plcR* mutant would not reach the Bt407WT strain virulence, even after a longer infection time in our model.

Several hypotheses could explain that spores are more virulent than vegetative cells to *G. mellonella* when inoculated by injection. When bacteria reach the haemocoel, they face the basic innate cellular and humoral immune responses of their host. While the vegetative form can be directly damaged by antimicrobial peptides or proteins, such as lysozyme, gallerimycin, or gloverin [25], the spores are not affected until germination by these molecules. Another response of the host lays in the production of phenol oxidase and clotting factors, which should act faster towards vegetative bacteria through their liberation of PAMP (pathogen-associated molecular pattern) than towards spores [26,27]. Therefore, host responses might be different at a later time point of infection, or the spores possess a factor that blocks part of the immune response. On the other hand, spores might be more easily phagocytosed by specialized hemocytes and could, thus, be damaged [25,28]. Meanwhile, according to a study using mammalian phagocytes, spores of Bt407WT were able to geminate and multiply inside macrophages [29], and Dubovskiy et al. [28] showed phagocytosis of a vegetative *B. thuringiensis* strain following oral infection of *G. mellonella*, but no information related to spore versus vegetative bacteria was reported in that paper. Another hypothesis to explain the greater virulence of spores is that they could constitute a reservoir of continuously released vegetative cells, which at the end would overcome host defenses. Alternatively, spores could concentrate, through its adhesion capabilities, on specific tissues, which would then be strongly damaged by secreted virulence factors after germination. A histopathological study designed to follow the fate of spores in the larval body could help distinguish between the different hypotheses.

Our results are in disagreement with conclusions of a previous study, in which the PlcR virulence regulon was found to have no effect on bacterial virulence when injected into the haemocoel of *G. mellonella* [13]. However, in our study, both spores and vegetative bacteria from the *∆plcR* mutant are about five-fold less virulent than Bt407WT at both temperatures. Perhaps in the former assays the number of repetitions and doses were lower, although the LD_50_ was determined by Probit. Here we used several doses and determined the LD_50_ by a statistical method (non-linear regression), which takes into account all doses for the LD_50_ calculation. In addition, our results are supported by other studies; for instance, the supernatant from the ∆plcR mutant was less cytotoxic to *Galleria* hemocytes than the supernatant from the Bt407WT culture [13], and some genes from the PlcR regulon are expressed in the haemocoel of *G. mellonella* (*plcB, mpbE*) [9,12], although expression does not prove a role in infection and virulence. The role of PlcR in *G. mellonella* infected by the oral route was previously shown to be strong, since spores of the *∆plcR* mutant killed only 10% of the larvae, while the same dose of spores from the wild-type strain killed 70% of the larvae [13]. In a similar study, one of the PlcR-regulated factors, the metalloprotease InhA2 was shown to have a major role in virulence [30] after oral infection. Additionally, other PlcR-regulated genes were shown to be important for *B. thuringiensis* virulence against insects by ingestion, including a collagenase [31] or the Enhancin-like Bel protease [32]. In contrast, the role of PlcR in *B. thuringiensis* virulence against the nematode *Caenorhabditis elegans* after ingestion of bacteria seems to be weak [33]. Therefore, we have actually more clues on the implication of individual genes from the PlcR regulon following oral infection than by haemocoel infection. Further studies are needed to identify factors of the PlcR regulon that play major roles in the haemocoel; this issue should be investigated in correlation with the innate cell and humoral host responses. 

Our study shows that environment-resistant spores of *B. thuringiensis* are also the most efficient at infecting *G. mellonella*, even in the haemocoel. At present, the study is conducted in the laboratory with relatively high doses. It will be interesting to set up experiments in more natural conditions and with other insects and strains. Indeed, for instance *G. mellonella* is much less susceptible to Bt407WT than *Bombyx mori*, for which a very small amount of bacteria introduced into the haemocoel kill large fifth instars [34]. Likewise, it would be important to test the outcome of spores versus vegetative bacteria in *Galleria* infected with a crystal producing strain active to this insect [25]. 

As mentioned in the introduction it is triggering that, for several *B. thuringiensis* strains, the spores and toxin crystal dissociate after mother cell lysis, while other *B. thuringiensis* strains are reported to keep the inclusion body inside the exosporium [35]. Whether it is the case for the strain naturally toxic to *G. mellonella* [25] or those reported in Li et al. 1987 [7] remains to be studied. In addition, the relative role of the PlcR–PapR quorum sensing systems [15] was analyzed from an ecological issue in *Plutella xylostella* (Diamondback Moth) [36]: in this insect, results also indicated that this system is important to fulfil infection and sporulation. Altogether our study contributes further to the role of the PlcR regulon in insect virulence and raises questions on the importance of spores in infection from the cuticle site for *B. thuringiensis* persistence and transmission. 

## 5. Conclusions

We studied the role of two infective forms of *B. thuringiensis* following injection into the haemocoel of *G. mellonella*. Our results show that by this infection route i) spores are more virulent than vegetative bacteria, and ii) factors controlled by the PlcR regulator are involved in pathogenesis and larval mortality. This indicates that the common virulence factors of *B. thuringiensis* and *B. cereus* strains may take part in pathogenesis both through the cuticle and following ingestion. These findings offer valuable perspectives for the development of ecological and epidemiological models of *B. thuringiensis* infections.

## Figures and Tables

**Table 1:** 50% Lethal dose (LD_50_ values) and 95% confidence intervals (CI_95%_) of Bt407WT or ∆*plcR* mutant strains estimated from mortality data following infection with spores.

Temperature	Strain	LD_50_ *	[CI_95%_]	Ratio *∆plcR* Mutant v.s. Bt407WT
37 °C	Bt407WT	1261	[606–1757]	
	∆*plcR* mutant	7344	[5871–9050]	5.8
25 °C	Bt407WT	2088	[1391–2873]	
	∆*plcR* mutant	9858	[1015–7734]	4.7

* LD_50_: Dose of spores per larva, killing 50% of infected individuals.

**Table 2:** 50% Lethal dose (LD_50_ values) and 95% confidence intervals (CI_95%_) of Bt407WT or ∆*plcR* mutant strains estimated from mortality data following infection with vegetative bacteria.

Temperature	Strain	LD_50_	[CI_95%_]	Ratio *∆plcR* Mutant vs. Bt407WT
37 °C	Bt407WT	5148	[3618–7087]	
	∆*plcR* mutant	19,213	[17,237–25,689]	3.7
25 °C	Bt407WT	4032	[2981–5260]	
	∆*plcR* mutant	21,395	[16,789–29,146]	5.3

* LD_50_: Dose of bacteria per larva, killing 50% of infected individuals.
